# The Clinical Impacts of Mobile Mood-Monitoring in Young People With Mental Health Problems: The MeMO Study

**DOI:** 10.3389/fpsyt.2021.687270

**Published:** 2021-07-30

**Authors:** Muna Dubad, Farah Elahi, Steven Marwaha

**Affiliations:** ^1^Mental Health and Wellbeing, Division of Health Sciences, Warwick Medical School, University of Warwick, Coventry, United Kingdom; ^2^Institute for Mental Health, University of Birmingham, Birmingham, United Kingdom; ^3^Specialist Mood Disorders Clinic, Zinnia Centre, Birmingham, United Kingdom

**Keywords:** mental health, mood, youth, ecological momentary assessment, technology, smartphone application, impulsivity

## Abstract

**Background:** Limited evidence suggests that mobile mood-monitoring can improve mental health outcomes and therapeutic engagement in young people. The aim of this mixed methods study was to explore the clinical impacts of mobile mood-monitoring in youth with mental health problems, using a publicly accessible app.

**Methods:** Twenty-three patients with mental health problems and 24 young people without mental health problems participated in the quantitative study. Participants monitored their mood using a mood-monitoring app twice a day for 3 weeks, which was preceded by a 3-week baseline period. Outcome measures included momentary and retrospective assessments of affect regulation (all participants) and therapeutic engagement (patients only). Following the quantitative study, patients (*n* = 7) and their clinicians (*n* = 6) participated in individual interviews. Interview data was analysed using thematic analysis.

**Results:** Use of the mood-monitoring app significantly reduced momentary negative mood (*p* < 0.001) and retrospectively assessed impulsivity across all 47 participants (*p* = 0.001). All other outcomes showed no significant difference. Qualitative feedback similarly indicated the potential of apps to improve problems with impulsivity in patients. Furthermore, apps may aid communication, promote empowerment, and ameliorate memory difficulties in clinical appointments.

**Conclusions:** This mixed methods study demonstrated the potential utility of apps for clinical practice. Apps may potentially be an interventional tool, or at a minimum, an adjunct to existing treatments. Data was collected from a small sample size over a short study duration, limiting the generalisability of findings and inferences regarding long-term effects. Potential sources of bias in the qualitative study (e.g., researcher bias) should also be considered.

## Introduction

Preliminary and limited evidence from ecological momentary assessment (EMA) studies indicates that mood-monitoring tools may improve mental health outcomes and therapeutic engagement in youth (1). Benefits may include increased self-awareness (2), which can (indirectly) improve young people's depressive symptoms (3). In their pilot study, Kinderman et al. (4) investigated the short-term impacts of the ‘Catch It’ app on users' moods. With each entry, app users: (1) rated the intensity of their positive or negative mood; (2) reflected on and cognitively appraised their mood by considering different perspectives; and (3) rated the intensity of their mood for a second time. On average, “Catch It” significantly increased app users' positive moods and significantly reduced negative moods from the first entry to the second entry. Although not tested in this study, this cognitive reappraisal strategy has been shown to reduce subjective, behavioural, physiological, and neural measures of emotional reactivity (5, 6).

Despite these encouraging findings, there are some weaknesses in the literature. A large proportion of EMA studies have either not taken advantage of smartphone apps (7, 8) or employed apps which are not publicly accessible (9). Previous studies also predominantly focused on adult populations, non-clinical populations, and/or specific diagnostic groups, particularly borderline personality disorder (9–11).

It is often assumed that young people in particular will embrace smartphones for the management of their mental health. Whilst these pre-conceived ideas may drive changes in digital health services, they are rarely tested and may not correspond with how young people use, perceive, and engage with technology in practice (12, 13). An online survey of 11–16 years old girls revealed that despite their high rates of Internet and app usage, only 15–17% of respondents with mental health problems had used a mental health app (12). Moreover, 22–24% of these respondents expressed preference for face-to-face appointments over apps, and 26–31% of respondents did not think an app would be helpful to them. Young people in this study reported various concerns about the use of mental health apps, such as apprehensions about the accuracy of information on the app, worries about privacy and unauthorised access, and a lack of trust in apps.

Although systematic review evidence suggests that apps are usable for young people, there is a need for qualitative studies to further examine young people' and clinicians' perceptions (13), both of which have not been sufficiently considered in the literature (1). Whilst studies (14, 15) suggest that healthcare practitioners are very interested in the integration of smartphone technology in treatment, actual uptake of, and familiarity with, apps is low. Lack of confidence with technology, and little guidance regarding the selection of apps, are some of the barriers that may affect healthcare practitioners' use of apps in mental health services (14).

A qualitative study by Terp et al. (16) described how features of a smartphone app, such as a medication overview and action planning, allowed young people with a recent diagnosis of schizophrenia to keep track of their mental health and progress, and enabled them to receive help based on their needs. Through these processes, the app helped young people to be in control of their condition, therefore empowering them. However, the efficacy of the app relied on the involvement of healthcare practitioners who helped alleviate some of the young people's concerns about the app. The successful implementation of smartphone technology in mental health services is therefore contingent upon the engagement of both service users and their healthcare providers. Thus, qualitative research methodology can be a powerful approach for exploring the views of both clinicians and patients in mental health settings (17).

In view of these limitations, further mixed methods research is needed to study the use and potential clinical impacts of publicly available app-based momentary assessment tools. The aim of this study was to investigate the clinical impacts of mobile mood monitoring in young people with mental health problems using quantitative and qualitative methods. Specifically, it examined the following research questions:

1) Does mobile mood-monitoring impact on momentary and retrospective measures of affect and engagement?
2) What are young patients and clinicians' views on the clinical and treatment impacts of mobile mood-monitoring?

## Methods

### Study Design

**Figure 1:**
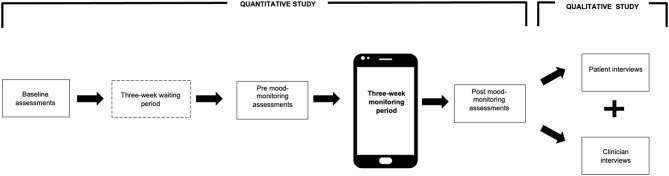
Overview of study design.

This study employed a mixed methods design, combining both qualitative and quantitative components. As seen in Figure 1, the quantitative study was conducted first, which employed a quasi-experimental pre-test—post-test design (18). This was followed by the qualitative study, which involved interviews with both patients and clinicians.

### Recruitment and Eligibility Criteria

This study was conducted as part a wider study on the use of digital mood-monitoring technology to support the assessment, engagement, and empowerment of young people presenting to mental health services with affective instability. Participants from the clinical group (aged 16–24 years) were recruited from a mental health charity (Mind) as well as UK National Health Service (NHS) based Child and Adolescent Mental Health Services and Adult Mental Health Services across the West Midlands. The study was advertised on posters (e.g., waiting rooms) and through social media (Facebook and Twitter) where further information and contact information was provided. In NHS services, mental health practitioners were also asked to identify eligible patients and tell them about the study. Eligible patients who expressed an interest were asked for their consent to release their contact details in order to receive further information. Of note, clinicians had no further involvement in the quantitative study. Patients therefore used the app for the purpose of the study as opposed to their standard treatment.

Young people (aged 16–24 years) from the healthy comparison group were primarily recruited via social media (Facebook, Instagram, and Twitter), however, recruitment posters were also displayed at university buildings. Prior to commencing the study, potential participants for this group were first required to confirm they met the eligibility criteria (e.g., “absence of current diagnosed mental disorder”) and subsequently completed a screening measure to exclude potential psychopathology. The eligibility criteria for both groups are listed in Table 1.

**Table 1:** Eligibility criteria for the clinical and healthy comparison group.

**Clinical group**	**Healthy comparison group**
1. Has capacity to consent (as assessed by the clinician and verified by Muna Dubad (MD)).	1. Aged 16–24.
2. Currently receives mental healthcare (i.e., not discharged).	2. Absence of current diagnosed mental disorder.
3. Aged 16–24.	3. No previous diagnosis of borderline personality disorder, bipolar disorder, psychosis, or Attention Deficit Hyperactivity Disorder (ADHD).
4. Has a psychiatric diagnosis.	4. No diagnosed learning disability.
5. Currently experiences affect, mood, or emotional instability or dysregulation, irrespective of diagnosis.	5. Not currently involved in other research.
6. No current need for inpatient or crisis team input.	6. Access to an iOS or Android (4.0 and up) smartphone.
7. No diagnosed learning disability.	7. Understands/speaks English (at a level sufficient enough to understand and complete questionnaires or diaries).
8. Not currently involved in other research.	
9. Access to an iOS or Android (4.0 and up) smartphone.	
10. Understands/speaks English (at a level sufficient enough to understand and complete questionnaires or diaries).	

Following the quantitative study, participants in the clinical group were invited to take part in an interview, which explored their experiences of using the mood-monitoring app. Clinicians who referred and were involved in the care of participants in the clinical group were also invited for interviews. Ethical approval for both studies was obtained from East Midlands Leicester Central Research Ethics Committee (reference: 17/EM/0146).

### Materials

#### Apps

The “Catch It” app was the selected mood-monitoring app for the study, based on feedback from a young person's steering group, students, and professionals, alongside an examination of the app features and security settings. In summary, the “Catch It” app consists of a multi-stage process (4). At the “Catch it” stage, app users are asked to rate their initial mood on a scale of 1–5 and describe the circumstances and thoughts associated with their mood or change in mood. At the “Check it” stage, the app helps users to reflect on what they are thinking. Finally, in the “Change it” stage, users are encouraged to consider different approaches. Following this consideration, users were asked to rate their mood a second time and were provided with brief, general feedback on their mood ratings [see Kinderman et al. (4) for further details]. In order to address the absence of a reminder feature in the “Catch It” app, participants downloaded a reminder app [“Randomly RemindMe” for Android users [James Morris (19)] and “Mind Jogger” (20) for iPhone users]. These apps prompted participants to complete a mood diary at two random times during the day (a time window of 10–12 h was typically chosen).

#### Measures

##### Screening Measure

Participants in the healthy comparison group were asked to complete the GHQ-12 screening measure (21) to determine their eligibility for the quantitative study. The GHQ-12 is a self-report questionnaire that detects the presence of psychopathology in community and non-psychiatric clinical settings. A score of four or higher suggested potential psychopathology (22, 23).

##### Outcome Measures

The primary outcome measures for the quantitative study was the Difficulties in Emotion Regulation Scale -Short Form [DERS-SF: (24)]. This measure was completed by all participants. Higher scores on the DERS-SF (i.e., total scores of all items within and across sub-scales) indicated more difficulties with affect regulation. The Engagement Scale (25) was used as a secondary outcome measure and was completed by patients only. Mean total scores for overall engagement were computed, in which higher scores reflected higher levels of engagement. The Affective Lability Scale [ALS-SF: (26)] was also used as a secondary outcome measure and was completed by all participants. Higher scores described increased shifts in affective states.

##### Demographic Data

Demographic information and GP details for NHS participants were accessible via CareNotes (an electronic patient database). Participants in the healthy comparison group, whose records were not electronically accessible, were asked to complete a form asking for demographic information and GP details.

##### Semi-Structured Interview Schedule

Semi-structured interview schedules for the qualitative study were derived from a topic guide (27), which included topics such as the ease of use of the app for patients and the perceived utility of the app data for clinicians. Interview schedules were developed for patients (10 questions) and clinicians (eight questions).

### Procedure

The quantitative mood-monitoring study was conducted in distinct stages, which was identical for both groups. First, all eligible participants provided written consent and were given support (e.g., with downloading apps). They then completed the study questionnaires and demographic/GP information form (where applicable). After a 3-week waiting period, all participants were prompted to complete the same questionnaires. Each participant then started the 3-week mood-monitoring period using the app (twice daily). Following this period, all participants sent the mood-monitoring data using the in-app export function, after which they completed the questionnaires for the final time. Each participant received a gift voucher upon completion (maximum £25, including reimbursement for travel expenses).

Participants from the clinical group and clinicians who expressed an interest in the qualitative study were invited to face-to-face or telephone interviews with MD, depending on individual needs and preferences. All participants provided written consent. Patients received a £10 gift voucher following interviews. Clinicians' contributions were acknowledged in personalised certificates and an accompanying letter. Audio recordings were stored on a password-protected computer at the University of Warwick and transcribed using Appen's (28) transcription service.

### Analyses

*Research question 1: Does mobile mood-monitoring impact on momentary and retrospective measures of affect and engagement?*

#### Momentary Outcomes

Positive and negative mood intensity ratings were analysed separately (4). Average mood intensity scores were first calculated for each individual in *Microsoft Excel*. A mixed analysis of variance (ANOVA) was subsequently conducted using *SPSS*. This analysis assessed whether there was a significant main effect for time (i.e., within group differences in average moods over time across all participants), and whether there was a significant interaction effect between the group (clinical and healthy) and time variable. In line with Kinderman et al. (4), findings were confirmed using a repeated measured mixed model. This multilevel model accounts for the multiple assessments per participants by adding a random effect for the ID variable (4). This helps illustrate the unique variations in mood intensity that can be attributed to individual differences (29).

Of note, the severity of mood ratings in the “Catch It” app was automatically set to 1 (4). If users selected a different mood intensity rating on the first entry but did not actively rate their mood on the second entry (i.e., leaving it at “1”), this could lead to false conclusions about the direction of results (see “Discussion”). Consistent with Kinderman et al. (4), a second, more conservative, analysis was performed to account for this, which excluded data in which the second mood rating post-reflection was 1.

#### Retrospective Outcomes

Mixed ANOVAs were conducted to assess the impact of the “Catch It” app on retrospective measures of affect across three time points (time 1: baseline assessments, time 2: pre mood-monitoring study assessments, time 3: post mood-monitoring study assessments). This included “emotion regulation,” which was measured as the total DERS-SF score, containing the sum of all items, as well as “emotional awareness,” “emotional clarity,” and “impulsivity,” which were derived from their respective DERS-SF sub-scales, containing the sum of three items per sub-scale. The final measure of affect was “shifts in affective states,” which was measured as the ALS-SF total mean score. A one-way repeated measures ANOVA was conducted to assess differences in patients' engagement. Engagement was measured as the mean total Engagement scale scores across the three time points. Paired-sampled *post-hoc t*-tests were conducted if significant main effects were found. Interaction effects were examined to establish whether effects of the app applied to all participants or varied across groups.

A Bonferroni adjusted alpha level of 0.005 was used to determine statistical significance across all significance tests.

*Research question 2: What are young patients and clinicians' views on the clinical and treatment impacts of mobile mood-monitoring?*

Interview data was analysed using Braun and Clarke's (30) thematic analysis method. This widely employed qualitative research method is not restricted to a particular epistemological or theoretical framework, and enables researchers to systematically identify key themes, in large amounts of data acquired from multiple participants (30, 31). On receipt, MD familiarised herself with the data by listening back to interview recordings. Transcripts were read and re-read and corrected for transcription errors where necessary. MD developed initial codes and themes. Farah Elahi (FE) separately coded ~50% of anonymous transcripts. MD assessed FE's codes against her codes to assess their validity (32) and further develop themes. The final themes were reviewed by all authors. All data was managed using *NVivo version 12* software.

## Results

### Descriptive Statistics

#### Sample Characteristics

**Figure 2:**
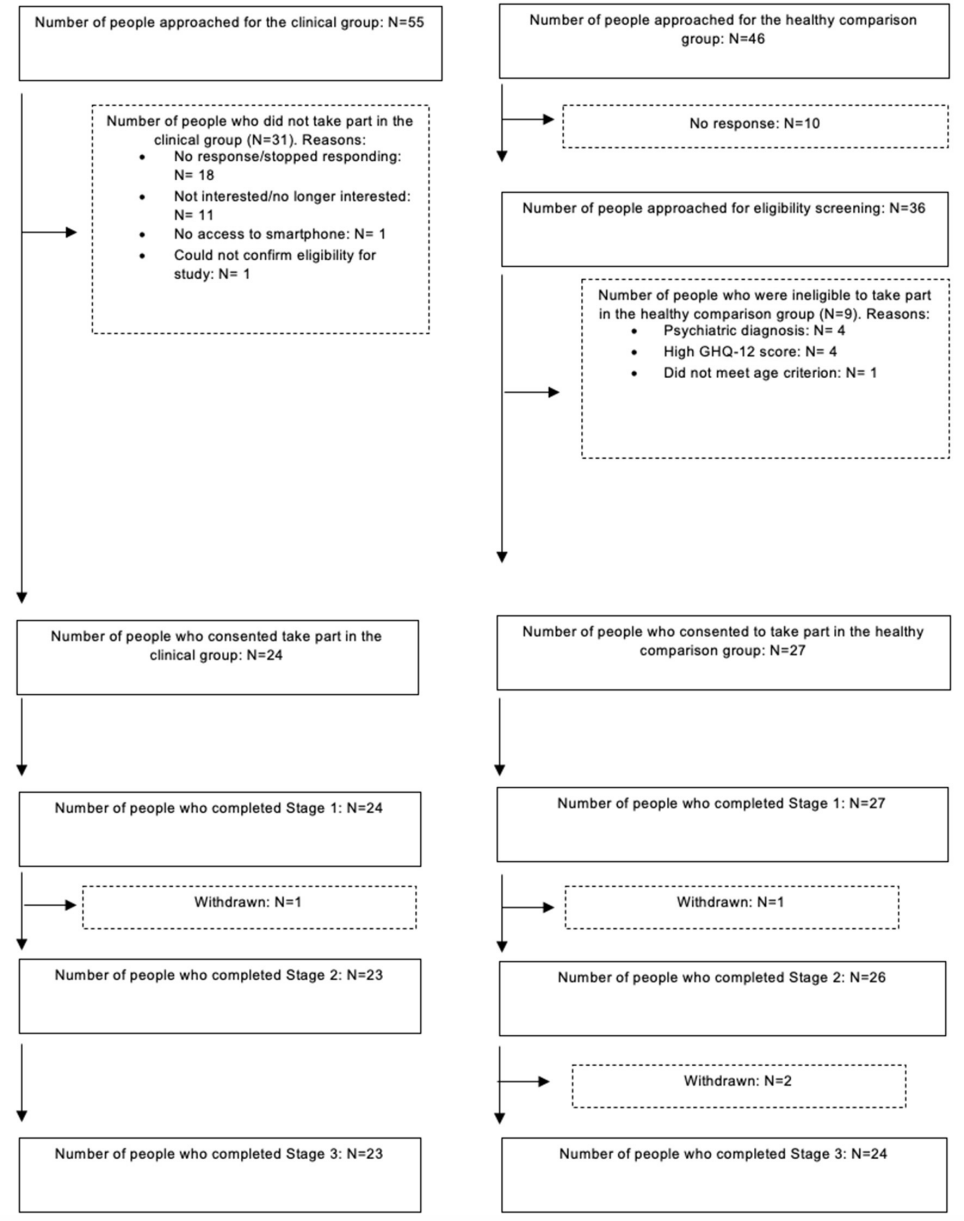
Overview of study recruitment process.

A total of 101 people were invited to the quantitative mood-monitoring study. They were allocated to the clinical (*n* = 55) or healthy comparison group (*n* = 46). In the clinical group, 24 participants provided consent, of which one participant withdrew due to personal circumstances and competing demands at school. In the healthy comparison group, 27 eligible people provided consent, of which one participant withdrew due to competing demands at work and two were lost-to-follow up for unknown reasons. The final sample consisted of 47 participants, including 23 people with mental health problems and 24 people without current mental health problems, with a mean age of 20.70 years [standard deviation (SD) = 3.17]. Table 2 describes the sample characteristics. The recruitment process is illustrated in Figure 2.

**Table 2:** Characteristics of the clinical and healthy comparison group.

**Characteristics**	**Clinical group**	**Healthy comparison group**	***p***
Age in years, mean (SD)	20.13 (2.9)	21.23 (3.4)	0.22
Gender, *n* (%)			1.00
Female	11 (47.8)	12 (50.0)	
Male	12 (52.2)	12 (50.0)	
Ethnicity, *n* (%)			0.10
White British	12 (52.2)	11 (45.8)	
Other white background	1 (4.3)		
Black African		4 (16.7)	
Other black background	1 (4.3)		
Indian		1 (4.2)	
Pakistani		4 (16.7)	
Other asian background	1 (4.3)	3 (12.5)	
Black and white heritage	2 (8.7)		
Other ethnic background		1 (4.2)	
Not recorded/available	6 (26.1)		
Employment status			
Employed		7 (29.2)	
Not in education, employment, or training		1 (4.2)	
In education/learning		16 (66.7)	
Not recorded	23 (100.0)		
Medication, *n* (%)			
On medication	19 (82.6)		
Not on medication	4 (17.4)		
Not applicable/not requested		24 (100.0)	
Diagnoses, *n* (%)			
Psychotic disorders with/without comorbidity	6 (26.1)		
Mood, panic, eating, and/or anxiety disorders with/without comorbidity	11 (47.8)		
(Emerging) personality disorders with/without comorbidity	6 (26.1)		

Out of the 23 patients who participated in the quantitative study, five declined to be contacted for the qualitative study and 12 initially expressed an interest, but subsequently declined (*n* = 1) or did not respond to interview invitations (*n* = 10). The final clinical sample for the qualitative study therefore consisted of seven participants (three females and four males), aged 17–24 years (Mean age = 20.71, SD = 2.56).

Thirteen clinicians were approached for the qualitative study, of which six responded to interview invitations and participated in the interviews. The clinician sample comprised two consultant psychiatrists, three community psychiatric nurses, and an assistant practitioner in mental health. On average, clinicians worked in their respective roles for 8.5 years (SD = 7.4, range = 1.5–21 years).

### Research Question 1: Does Mobile Mood-Monitoring Impact on Momentary and Retrospective Measures of Affect and Engagement?

#### Momentary Clinical Outcomes

There was a significant reduction in the intensity of negative mood across all analyses and participants (*n* = 47) which included all valid recordings (i.e., recordings which were interpretable and had both pre- and post-values), *F*_(1,45)_ = 41.83, *p* < 0.001, and those which excluded potentially confounding recordings, *F*_(1,45)_ = 14.82, *p* < 0.001. In contrast, there was no significant improvement in the intensity of positive mood over time across all analyses and participants (*n* = 47) using the Bonferroni corrected threshold of *p* = 0.005 (see Table 3). There was no significant interaction between “time” and “group” in all analyses, indicating effects did not vary across groups (range: *p* = 0.58–0.82).

**Table 3:** Positive and negative mood intensity ratings.

**Analysis**	**Rating**	**Positive mood intensity**		**Negative mood intensity**
All valid recordings^a^	First entry, mean (SD)	3.18 (0.84)	*p =* 0.016^b^	2.97 (0.81)^*^
	Second entry, mean (SD)	2.83 (0.91)		2.32 (0.85)
Exclusion of potentially confounding default recordings	First entry, mean (SD)	3.32 (0.60)	*p =* 0.017^b^	3.42 (0.62)^*^
	Second entry, mean (SD)	3.50 (0.62)		2.95 (0.68)

a *Recordings which were interpretable and had both pre- and post-values*.

* *Significant difference at p < 0.005 (Bonferroni corrected threshold) from the first to the second entry*.

b *Difference from the first entry to the second entry was determined to be non-significant using the Bonferroni corrected threshold*.

#### Retrospective Clinical Outcomes

There was a significant main effect for time for the “Impulse” subscale, Wilks Lambda = 0.74, *F*_(2,44)_ = 7.69, *p* = 0.001, partial eta squared = 0.26. This suggests there was a significant decrease in impulsivity over time across all participants (*n* = 47). There was no significant interaction between “time” and “group”; Wilks Lambda = 0.98, *F*_(2,44)_ = 0.55, *p* = 0.58, indicating that the significant effect did not vary across groups. A *post-hoc* test revealed no significant difference between time 1 and time 2 (*p* = 0.69) and between time 2 and time 3 (*p* = 0.02; Bonferroni corrected threshold: *p* = 0.005). There was a significant reduction between time 1 and time 3 (*p* < 0.001).

There was no significant main effect for time for any of the other DERS-SF retrospective outcomes, including: total emotion regulation difficulties (*p* = 0.11), emotional awareness (*p* = 0.82), and emotional clarity (*p* = 0.68). All interaction effects were non-significant (range: *p* = 0.42–0.77). Mean scores for the total and DERS-SF subscales are presented in Table 4.

**Table 4:** Overview of DERS-SF total and sub-scale scores.

	**Total**		**Sub-scales**	
**Time**	**Emotion regulation**	**Clarity**	**Awareness**	**Impulse**
1	46.77 (14.19)	7.47 (2.70)	7.68 (3.20)	6.64 (3.38)^*^
2	46.70 (16.39)	7.17 (3.12)	7.85 (3.03)	6.51 (3.62)
3	44.79 (15.92)	7.30 (3.19)	7.94 (2.79)	5.74 (2.94)^*^

*DERS-SF total score (scale = 1–90) and subscales scores (scale = 1–15). Higher scores reflect more difficulty*.

* *Significant difference between Time 1 and Time 3, p < 0.005 (Bonferroni corrected threshold)*.

There was no significant main effect for time for the ALS-SF mean total score, indicating no significant difference in affective shifts over time 1 (M = 1.24, SD = 0.72), time 2 (M = 1.21, SD = 0.73), and time 3 (M = 1.24; SD = 0.76); Wilks Lambda = 0.98, *F*_(2,44)_ = 0.49, *p* = 0.62.

Finally, there was no significant main effect for time for mean total Engagement, suggesting no significant difference in patients' engagement across time 1 (Mea*n* = 2.91, SD = 0.56), time 2 (Mea*n* = 2.84, SD = 0.53), and time 3 (Mea*n* = 2.89, SD = 0.74); Wilks Lambda = 0.97, *F*_(2,21)_ = 0.32, *p* = 0.73.

### Research Question 2: What Are Young Patients and Clinicians' Views on the Clinical and Treatment Impacts of Mobile Mood-Monitoring?

Two cross-cutting themes from patient and clinician interviews were identified. Quotes were reported verbatim in quotation marks.

*Theme 1—Communication, memory, and implications for treatment*

There was some indication that apps may positively influence communication with clinicians. The app encouraged one patient to communicate more honestly with his clinician. For some patients, the app also facilitated communication with friends, family and significant others. For example:

“It, kind of, made it easier to talk to, like, friends and family, which obviously helps,” because, “rather than just trying to explain to them how I'm feeling, I could just show them and then we'll talk about it, rather than just, well, attempting to explain.” [Participant ID 2, female aged 23]

Several patients described difficulties with recalling their emotions during appointments, and the way in which the app could have or had helped. For instance:

“.sometimes, like, I forget, like, what I get anxious about [.] and it's just easier just to show them. So they can, like, kind of, like, understand it a bit better.” [Participant ID 1, female]

Similarly, some clinicians discussed difficulties in obtaining information from young patients in clinics, which were partly attributed to difficulties in remembering details during appointments, and could be ameliorated through apps:

“.sometimes we, or the, the service users struggle to remember what happened last week or 2 days ago. So this would be a good way of monitoring, having it on there. And it's a, I think for them it would be a nice, cool way to do that.” [Clinician ID 6, Psychiatric Nurse]

Patients expressed reservations about the utility of apps in terms of their impact on care and treatment engagement. Only one patient thought the app helped him feel more engaged with his treatment, stating that the app helped him keep track of what he was feeling at the time. Another patient felt the app had potential to improve engagement, depending how it is applied.

Finally, clinicians highlighted opportunities for collaboration and reflection in treatment, such as the use of mood-monitoring data to inform relevant coping strategies and relapse prevention plans:

“It builds my relationship with them because I could turn around and say, “Let's look at it together,” kind of a thing. So, and we are not just going by their word. [.] And I'm not just showing my interpretation. [.] So some, somebody else's random analysis or list is there. [.] So it might actually, they might actually feel that this person is not saying it, it's me who's done it actually.” [Clinician ID 4, Consultant Psychiatrist]

*Theme 2—Reflection, self-awareness, and affect regulation*

Several patients discussed whether and how the app aided reflections on their mood. For some patients, the experience was positive, with feedback describing the usefulness of labelling moods and the increased understanding of underlying causes of moods:

“[it was] really useful to, to, sort of put a, a label on how you're feeling” [Participant ID 16, male aged 22]

Patients also appreciated the app's ability to provide an emotional outlet, as opposed to holding onto their thoughts in their minds:

“When I didn't [use the app], it was almost like I had the world on my shoulders. A heavy weight and stuff like that. Like, the, the, the proper cliché sort of metaphors of having a lot of things on your mind. But then, like, when I was using the app, there was a lot of those ones that were going down onto the, the page. And so it was lifting quite a heavy weight off. And then you can sort of carry on.” [Participant ID 4, male aged 17]

Importantly, some patients also noticed a reduction in impulsive or reactive behaviours, as well as an increased ability to self-manage moods:

“It stopped me cutting, which was good because I managed to circumvent it by taking the 5 min out and doing, and doing an entry before or, yeah, just before I even felt like I needed to. So it stopped me.” [Participant ID 4, male aged 17]

Another appeared to have felt more empowered through the app. She noted:

“…when I was, like, talking to, like, I forgot what her name is, like, I guess I couldn't, like, remember it all, like, the emotions I have. […] And then afterwards, like, like, with the app, like, I kind of, like, knew them, and I kind of could deal with it myself, like. I feel like I was, like, relying on, like, other people more than, like […] myself. And when I used the app like I was relying on myself a bit more. […] I mean, obviously it's nice to have people to help, but, like […] in, like, like, you know. to be honest with you, like, most of the time you've only really got yourself.” [Participant ID 1, female aged 18]

Clinicians similarly valued the app's potential to strengthen their understanding of young people's difficulties. For instance, one clinician developed a new perspective on one of her patients' eating problems upon reviewing his data:

“…you can make links between his eating problems and his moods. So, e.g., he felt anxious, that's when he started eating, and then that reflects on his depression. So that's a circle that, you know, the cycle of emotions that he goes through. And if we can tackle his anxiety, perhaps we can tackle his eating a bit better and his moods a bit better. But this is the first time I've sort of seen it and connected the dots, and I wonder if he connected the dots for him whether he'd benefit from this as well. So, I think it's very, very useful from so many aspects.” [Clinician ID 5, Consultant Psychiatrist]

Notwithstanding this, some patients did not report an increased awareness and/or understanding of their moods. Moreover, some patients did not perceive the app as useful as it could have been in its current format and reported little to no change in their ability to regulate or control their mood and mental health.

## Discussion

This study aimed to investigate the clinical impacts of mobile mood monitoring in young people with mental health problems using mixed methods.

Mixed findings were reported regarding the impact of mood-monitoring on momentary and retrospective measures of affect and engagement. Contrary to Kinderman et al.'s (4) findings, use of the “Catch It” app did not significantly improve momentary positive moods across all participants in the current study, i.e., a (non-significant) reduction in positive moods was found when all ratings were included. However, the removal of potentially confounding ratings showed a (non-significant) increase in positive moods across groups. Notwithstanding this conflicting finding, momentary negative mood intensity scores significantly reduced for both groups, irrespective of the inclusion or exclusion of potentially confounding mood recordings. Given the link between negative affect and psychopathology (33), future endeavours should further examine momentary affect in youth through apps.

The only retrospective outcome which showed a significant improvement over time was “impulsivity.” Both groups showed a significant reduction in impulsivity from the start of the study compared to the end of the study. Hence, clinical and non-clinical populations can successfully use cognitive reappraisal strategies to reduce impulsivity. Gruber et al. (6) hypothesised that whilst people with mental health problems are able to efficiently regulate their emotions through cognitive reappraisal when prompted (e.g., via apps), they may struggle to apply these strategies in everyday life when unprompted. Patients may also not engage in cognitive reappraisal as frequently or as effectively as healthy individuals (6, 34). As impulsivity is associated with adverse outcomes, such as suicidal behaviours (35), the importance of supporting young people with using cognitive reappraisal strategies is highlighted (6). As “Catch It” had a self-monitoring feature and encouraged the use of cognitive reappraisal skills, both of which can positively influence behavioural or clinical outcomes (36–38), future studies should further dissect the individual and combined contributions of each skill on outcomes.

Self-reported engagement did not significantly improve over time. Patients used the app for the purpose of the study as opposed to their standard treatment. There was therefore a lack of direct clinician involvement in the study who monitored or reviewed app usage. As the therapeutic alliance is imperative for successful treatment outcomes (39) and because the efficacy of apps may be influenced by the direct involvement of clinicians (16), this may explain the lack of change in engagement (40).

Patients and clinicians' perceptions on the impacts of mobile mood-monitoring similarly varied. Overall, qualitative feedback indicated that the use of mood-monitoring apps may have important clinical and treatment benefits for young patients and clinicians. The act of self-monitoring and labelling emotions, for example, helped some patients develop a greater understanding and awareness of their mood. Previous studies suggest this increase may improve mental health outcomes (3). Moreover, use of these app-based technologies may encourage patients to use effective self-regulation strategies to effectively manage their mental health (16). Indeed, some patients reported an improved ability to safely and independently manage their moods through the app. This suggests apps can promote patient empowerment, which subsequently could improve patient outcomes and experiences (41).

Consistent with findings from the quantitative mood-monitoring study, young patients experienced a reduction in impulsive or reactive behaviours as a result of using the app. This may be attributed to the aforementioned “Change It” feature of the app, which encouraged users to consider other perspectives (4). This finding further stresses the importance of supporting young people with using cognitive reappraisal strategies, which could help them better manage their affective experiences (6).

Feedback from patients and clinicians highlighted patients' difficulties with discussing, recalling, and estimating their moods over time (42). The app, through its capacity for EMA, could help patients overcome some of these difficulties by enabling real-time mood recordings (43, 44). This helped patients to more easily, and potentially more honestly, communicate information about their moods by showing clinicians their diary data. Moreover, it facilitated patients' communication within their day-to-day lives.

### Strengths and Limitations

This appears to be the first mixed methods study which investigated the usability and clinical impacts of a publicly accessible app. Although the study produced clinically important findings and discussed potential implications for practice, several limitations need to be acknowledged.

The quantitative study had a relatively small sample size and short study duration. This may have made it more difficult to capture meaningful differences or changes across outcomes. Future studies should also investigate whether the results can be replicated in patients with more acute levels of mental illness and sustained in the long-term.

It should also be noted that evidence for the sensitivity and specificity of the GHQ-12 screening measure varies across studies (45). Whilst participants' self-reports and GHQ-12 scores reduced the likelihood of diagnosable psychopathology in this group, it is nonetheless possible that some participants from the healthy comparison group potentially experienced low levels of mental health problems, such as depression and anxiety. This may have therefore affected the comparability of groups.

The app's aforementioned default rating of “1” potentially affected the interpretation of findings. For example, if a participant rated their “depression” as five on the first entry, but did not actively rate their mood on the second entry (i.e., leaving it at “1”), this would suggest a substantial reduction in depression from 5 to 1 (even though there may have been no improvement in reality). As such, the default rating could result in misleading conclusions about the effects of the app (4).

As previously mentioned, the lack of direct involvement of patients' clinicians in the study could have potentially influenced the efficacy of the app (e.g., the impact on engagement), which is therefore a limitation of the study.

With regard to the qualitative study, the majority of participants invited to interviews either declined to participate or did not respond to invitations. Given the predominantly positive feedback, the final sample was possibly biassed toward people with more favourable attitudes or experiences. A larger study using a purposive maximum variation sampling strategy can help diversify the group of participants and uncover this issue (16).

Another limitation pertains to the issue of data saturation. Limited and inconsistent guidelines are available to help researchers determine whether data saturation has been reached (46). Nevertheless, some studies (46, 47) suggested a sample size of 6–12 interviews to reach saturation. The current sample size fell within this range, and no new themes appeared to emerge from the final interviews. However, ideally (e.g., if there were more resources) a larger sample would have been used.

Finally, MD was responsible for data collection in both studies. Although MD's involvement helped establish initial rapport with participants, at an unconscious level, this may have introduced researcher and/or participant bias. Findings should be interpreted with these considerations in mind.

### Clinical and Research Implications

This mixed methods study demonstrated the potential utility of apps for clinical practice. For example, apps may help overcome patients' difficulties with memory recall and facilitate clinical communication. Moreover, they have the potential to improve clinical symptoms and increase patient empowerment. This suggests that apps may potentially be an interventional tool, or at a minimum, could be considered as an adjunct to existing treatments, albeit for young people with milder levels of mental health problems. This is particularly significant given the impact of the COVID-19 pandemic on mental well-being and the delivery of mental health services (48). Future studies should investigate: (1) at what stage apps are most effective (e.g., prevention); (2) whether findings can be replicated in patients with more severe psychopathologies; (3) whether benefits can be sustained in the long-term; (4) what specific features of the app contribute to psychological changes; and (5) whether improvements can be attributed to individuals' expectations of apps. This phenomenon, coined the digital placebo effect, is an overlooked area which also merits future investigation (49).

## Data Availability Statement

The datasets presented in this article are not readily available because informed consent was not obtained to publicly share the dataset. Requests to access the datasets should be directed to Leicester Central Research Ethics Committee, leicestercentral.rec@hra.nhs.uk.

## Ethics Statement

The studies involving human participants were reviewed and approved by Leicester Central Research Ethics Committee. The patients/participants provided their written informed consent to participate in this study.

## Author Contributions

MD: conceptualisation, methodology, formal analysis, investigation, project administration, and writing—original draft. FE: formal analysis and writing—review and editing. SM: conceptualisation, methodology, writing—review and editing, supervision, and funding acquisition. All authors contributed to the article and approved the submitted version.

## Conflict of Interest

The authors declare that the research was conducted in the absence of any commercial or financial relationships that could be construed as a potential conflict of interest.

## Publisher's Note

All claims expressed in this article are solely those of the authors and do not necessarily represent those of their affiliated organizations, or those of the publisher, the editors and the reviewers. Any product that may be evaluated in this article, or claim that may be made by its manufacturer, is not guaranteed or endorsed by the publisher.
